# Glycemic and Satiety Response to Three Mexican Honey Varieties

**DOI:** 10.3390/foods12193670

**Published:** 2023-10-06

**Authors:** Brenda A. Palomo-de León, Heriberto Castro, Mayra E. Sánchez-Murillo, Ana Laura de la Garza, Beatriz A. Rodríguez-Romero, Maritza Alonzo-Macías, Aurea K. Ramírez-Jiménez, Anaberta Cardador-Martínez, Marcelo Hernández-Salazar

**Affiliations:** 1 Centro de Investigación en Nutrición y Salud Pública, Facultad de Salud Pública y Nutrición, Autonomous University of Nuevo León, San Nicolás de los Garza 66455, Mexico; nut.brendapalomo@gmail.com (B.A.P.-d.L.); heriberto.castrogr@uanl.edu.mx (H.C.); msanchezmu@uanl.edu.mx (M.E.S.-M.); ana.delagarzah@uanl.edu.mx (A.L.d.l.G.); 2 Laboratorio de Evaluación Sensorial y Desarrollo de Nuevos Productos, Autonomous University of Nuevo León, San Nicolás de los Garza 66455, Mexico; beatriz.rodriguezrm@uanl.edu.mx; 3 Escuela de Ingeniería y Ciencias, Tecnologico de Monterrey, Queretaro 76130, Mexico; malonzoma@tec.mx (M.A.-M.); aramirezj@tec.mx (A.K.R.-J.); mcardador@tec.mx (A.C.-M.)

**Keywords:** glycemic index, satiety response, highland honey, avocado honey

## Abstract

Honey is considered one of the last untreated natural food substances, with a complex composition. It is produced by bees (*Apis mellifera*) from nectar. The glycemic index (GI) is a physiological assessment of a food’s carbohydrate content via its effect on postprandial blood glucose concentrations. This study evaluated the GI and the satiety response to three Mexican types of honey administered to 26 healthy volunteers. The fructose values ranged from 272.40 g/kg to 395.10 g/kg, while the glucose value ranged from 232.20 g/kg to 355.50 g/kg. The fructose/glucose (F/G) ratio of honey was 1.45, 1.00, and 1.17 for highland, multifloral, and avocado honey, respectively. Highland and avocado honey were classified as medium-GI (69.20 ± 4.07 and 66.36 ± 5.74, respectively), while multifloral honey was classified as high-GI (74.24 ± 5.98). Highland honey presented a higher satiety values response than glucose. The difference in GI values and the satiety response effect of highland honey could be explained by its different carbohydrate composition and the possible presence of other honey components such as phytochemicals. Honey, especially avocado, could therefore be used as a sweetener without altering significantly the blood glucose concentration.

## 1. Introduction

Honey is a natural substance produced by bees (*Apis mellifera*) from nectar, and it is considered one of the last untreated natural food substances. Mexico is the fifth-largest producer and the third-largest exporter of honey around the world; consequently, the beekeeping industry in Mexico has a high social and economic value [1,2].

Honey composition is complex and influenced by several factors, such as geographical origin, botanical source of nectar, and environmental and climatic conditions, as well as processing techniques, such as pasteurization or sterilization [3,4,5]. Botanical and geographical origins are considered the most important factors that determine the specific composition and properties of all types of honey [6]. According to their botanical origin, honey varieties can be classified as monofloral or multifloral. 

Meanwhile, the glycemic index (GI) is a physiological assessment of a food’s carbohydrate content via its effect on postprandial blood glucose concentrations. GI is measured as the incremental blood glucose area (0–2 h) usually following ingestion of 50 g of available carbohydrates as compared with 50 g of carbohydrates from a reference (glucose or white bread) [7]. Taking glucose as a reference, the values of GI could be classified as high (above 70), medium (56–69), and low (≤55) [8]. High-GI foods are considered unhealthy because their frequent consumption may increase the risk of developing overweight, obesity, type 2 diabetes, and cardiovascular disease [8,9].

The consumption of high-GI foods induces a rapid and high transient increase in postprandial glucose; conversely, the consumption of low-GI foods is associated with health benefits because they induce a lower blood glucose response [10]. A low-GI diet improves insulin sensitivity in obese children with high baseline insulin [11]. Thus, the effects exerted by the consumption of high-GI foods might negatively affect the satiety response, stimulating a proportionally high insulin response and inducing a low blood glucose and fatty acid concentration somewhat early in the postprandial period, mimicking a low-fuel state [12]. Nevertheless, it has been learned that foods with high-GI values might increase satiety and decrease hunger at different points in time in the postprandial period [7,8,13]. 

Honey has a lower GI and energy value than sugar as a result of its complex composition [6]. A mean GI value of 61 has been established for honey [14], although various factors could alter it, such as its carbohydrate composition and bioactive substances present in honey, such as phenolic compounds. Since these factors greatly vary depending on the botanical and geographical origin of honey, it is essential to conduct studies assessing the effect of those variations on the GI and other related properties.

Larson-Meyer et al. [15] indicated that as part of a meal, honey improves the postpandrial satiety response, increasing anorectic hormones, such as peptide tyrosine-tyrosine (PPY), and delaying orectic hormones such as ghrelin. Conversely, Gourdomichali and Papakonstantinou [16] did not find an effect on satiety from honey consumption. Despite the relevance of the beekeeping market, the GI and the potential satiety effect of Mexican honey have not been extensively investigated. The objective of this study was to evaluate the GI and the satiety response of three types of Mexican honey from different botanical and geographical origins.

## 2. Materials and Methods

### 2.1. Honey Samples

Three different types of Mexican honey from *Apis mellifera* with different geographical and flora origins were used to carry out the study: multifloral, avocado (*Persea americana*), and highland honey. The multifloral honey was harvested in Campeche State (southeastern region of Mexico); the avocado honey was harvested in Michoacan State, in the avocado-producing region, western region; and the highland honey was harvested in Mexico State (central highlands region). Honey samples were purchased from Hermes Honey (Aguascalientes, Ags., 20337, México), a company certificated by the True Source Honey Certification, and the European Union (https://hermeshoney.com/calidad/ (accessed on 25 September 2023)). Samples, which came from the 2019 harvest, were stored at room temperature and protected from light until analysis.

### 2.2. Fructose and Glucose Content by HPLC

An Agilent 1100 HPLC (Agilent Technologies, Palo Alto, CA, USA) was used to quantify fructose and glucose. An aliquot of 20 μL was injected into an Agilent Hi-Plex Ca 7.7 × 300 mm, 8 μm column at 85 °C with a flow rate of 0.6 mL/min. Pure water was used as an eluent. Detection was carried out in an Agilent Refractive Index Detector [17]. Fructose and glucose calibration curves (0–5 mg/mL) were used for quantification. All samples were injected in triplicate.

### 2.3. Study Design

This protocol was approved by the ethics committee of the Facultad de Salud Pública y Nutricion (FaSPyN) with the approval number CE 2/2018-19; in addition, all experimental procedures were conducted in accordance with the Declaration of Helsinki of 1975, revised in 2013, and all procedures involving human subjects were performed under the regulation of the General Law on Health Research. A cross-sectional study was carried out at Centro de Investigación en Nutrición y Salud Pública (CINSP) from the Autonomous University of Nuevo Leon, with residents of Monterrey, Nuevo León, México. All 18- to 40-year-old volunteers recruited were provided written informed consent prior to enrollment in the study. Inclusion criteria included: body mass index (BMI) ranging from 18 to 24.9 kg/m^2^ [18] and absence of chronic non-contagious diseases, which was corroborated via blood tests, including blood chemistry and glycosylated hemoglobin (HbA1c) tests. Participants with physiological conditions, such as pregnancy and lactation, and people with a different physical condition that impeded obtaining anthropometric parameters were excluded.

The participants arrived at the CINSP at 08:00 h for testing after a 12-h overnight fast. The study consisted of 4 testing sessions, to generate the glucose curves: one with an anhydrous glucose solution, and one more with each honey sample; there was a period of 5 days between each visit. Finally, participants consumed anhydrous glucose, or each honey sample diluted in 200 mL of water.

Before the study, fasting blood sugar (FBS), HbA1c, total cholesterol, high-density lipoprotein (HDL) cholesterol, low-density lipoprotein (LDL) cholesterol, and triacylgycerol were measured for each participant in the Departamento de Patología of the Hospital Universitario Dr. Eleuterio González. The anthropometric measurements were taken in the laboratory of the Centro de Investigación en Nutrición y Salud Pública of FaSPyN.

### 2.4. Glycemic Response

The GI of honey samples was evaluated using the proposed protocol by Jenkins et al. [19] with some modifications. Briefly, participants arrived at the laboratory under fasting conditions (at least 12 h) to be administered anhydrous glucose or honey. A blood sample was collected using capillary blood sampling via finger prick, and the blood sugar levels were taken as the baseline blood glucose concentration. Then, the participants consumed the honey test sample or anhydrous glucose within five minutes after the baseline sample had been collected. Capillary blood sugar was measured at 0, 15, 30, 60, 90, and 120 min. 0 min was considered the time participant ingested honey or anhydrous glucose for the first time. The procedure was repeated using 70–98 g of honey with 150 mL of water. The amount of honey administered provided 50 g of available carbohydrates. Capillary blood was analyzed immediately for blood glucose concentration using a calibrated Accu Chek Perfoma blood glucose meter (Roche Diagnostics, Pendzberg, Germany).

Then, the glucose concentration was plotted to calculate the area under the curve (AUC), considering 86 mg/dL as the baseline value. The AUC was calculated using Prism 5.0 software. This calculation was based on the trapezoid method. GI was calculated as the mean relation of honey AUC divided by dextrose AUC, expressing results as a percentage according to Equation (1).

GI = (Honey AUC)/(Glucose AUC) × 100(1)

### 2.5. Satiety Response

During the GI test, the satiety response was also evaluated. All participants answered the question “How hungry are you right now?” by indicating their satiety level on a visual analogous scale (VAS) [20]. Subjects rated hunger on a 100 mm line, anchored on the left by “not hungry at all” and on the right by “extremely hungry”. Measurements were taken during the generation of the glucose curve and the honey samples testing at 0, 15, 30, 45, 60, 90, and 120 min. The area under the curve of all rating values was determined, and the average values were calculated and plotted.

### 2.6. Statistical Analyses

Statistical analyses were conducted using the statistical software JMP version 9.0. Results are given as mean ± standard error of the mean. Differences in GI and satiety response among honey varieties were determined via analysis of Tukey’s HSD. Statistical significance was selected at a level of α ≤ 0.05.

## 3. Results and Discussion

Mexico is the tenth largest honey producer in the world [21]. Due to its wide biodiversity, it is a generator of honey with diverse physicochemical properties. There is no compilation of the total types of monofloral honeys in our country. However, in the Yucatan peninsula, the main exporting area, there is a record of more than 60 honeys with different colors [22], which means that the biological characteristics of these honeys can be diverse and of great interest. Therefore, it was decided to evaluate 3 representative honeys from different areas of the country; one from the central highlands (highland honey), another from the western zone (avocado honey) and one more from the Yucatan peninsula (multifloral honey), which would allow us to obtain a general image of the honeys produced in our country.

### 3.1. Fructose and Glucose Content

In this study, 3 different samples of honey, with different botanical and geographical origins, were analyzed.

Table 1 indicates the values of fructose and glucose as well as the ratio fructose/glucose (F/G). Honey samples presented fructose values ranging from 272.40 g/kg to 395.10 g/kg, while glucose values ranged from 232.20 g/kg to 355.50 g/kg. The results of fructose and glucose were statistically different between the honey samples (Table 1). The ratios F/G of honey samples were 1.45, 1.00, and 1.17 for highland, multifloral, and avocado honey, respectively.

Glucose and fructose were determined as available carbohydrates because they are the ones found in the highest concentration in honey. Monosaccharides represent about 75% of total carbohydrate content, of which approximately 40% is fructose [4]. After determination of glucose and fructose content, the available carbohydrate value per dose of honey was determined to be close to 50 g. To our knowledge, the fructose and glucose content of avocado and highland honey of Mexican origin has not been reported. In general, honey is 35–40% fructose and 30–35% glucose; in authentic honey, the F/G ratio is between 1 and 1.2 [23]. Sugar composition is affected by botanical origin, geographical origin, climate, processing, and storage [4,24]. Generally, fructose is the carbohydrate present in the greatest proportion [24], but in some types of honey, such as rape (*Brassica napus*) and dandelion (*Taraxacum officinale*) honey, the fraction of glucose is higher than the fraction of fructose; therefore, these honeys crystallize rapidly [4,25]. The concentration of fructose and glucose is used as an indicator for the classification of monofloral honey [24,25].

Fructose and glucose content have been determined in highland honey from Turkey at 409.1 and 275.60 g/kg, respectively, while the mean F/G ratio was 1.43 [26]; these results are similar to those found, in this study, in highland honey samples. In addition, the FAO has established the F/G ratio for flower honey is between 0.9 and 1.4 [27]. The F/G ratio found in this study for all honey samples agrees with the values reported by the FAO [27] (Table 1). On the other hand, the values of fructose and glucose found in multifloral honey samples were similar to those reported by Mondragón-Cortez, Ulloa, Rosas-Ulloa, Rodríguez-Rodríguez, and Resendiz Vázquez [2], who indicated values of 372.80 to 409.10 and 307.10 to 321.0 g/kg for fructose and glucose, respectively, in multifloral honey from the western region of Mexico.

Honey is an important natural sweetener due to its high simple sugar content (80%); however, it is healthier than sugar because it contains other important substances, such as phenolic compounds, which influence human health [5,6,28].

### 3.2. Anthropometry and Biochemical Data

Twenty-six participants completed this study. Table 2 shows the baseline conditions of all participants. The mean age of participants was 23.16 ± 3.73 years. The mean values of serum glucose and HbA1c were 84.29 ± 7.00 mg/dL and 5.38 ± 0.23%, respectively. Cholesterol, triglycerides, HDL, LDL, and VLDL showed mean values of 149.44 ± 19.96, 68.40 ± 29.88, 53.77 ± 12.65, 82.01 ± 17.22 and 13.07 ± 5.13 mg/dL, respectively. The mean weight of participants was 61.12 ± 11.69 kg, while the mean value of height was 1.62 ± 0.007 m; therefore, participants showed a mean value of BMI of 23.19 ± 3.07. These results were considered when choosing the participants to be included in the study.

### 3.3. The Glycemic Index (GI)

The values of GI and postprandial incremental glucose are shown in Table 3. The AUC values of honey samples were 155 ± 9.16, 169.06 ± 13.44, and 149.10 ± 12.90 for highland honey, multifloral honey, and avocado honey, respectively. Multifloral honey showed the highest AUC value, but it was not statistically significant concerning honey samples. However, all honey samples values were statistically significant compared to the glucose value (224.68 ± 18.48) (Table 3). 

**Figure 1:**
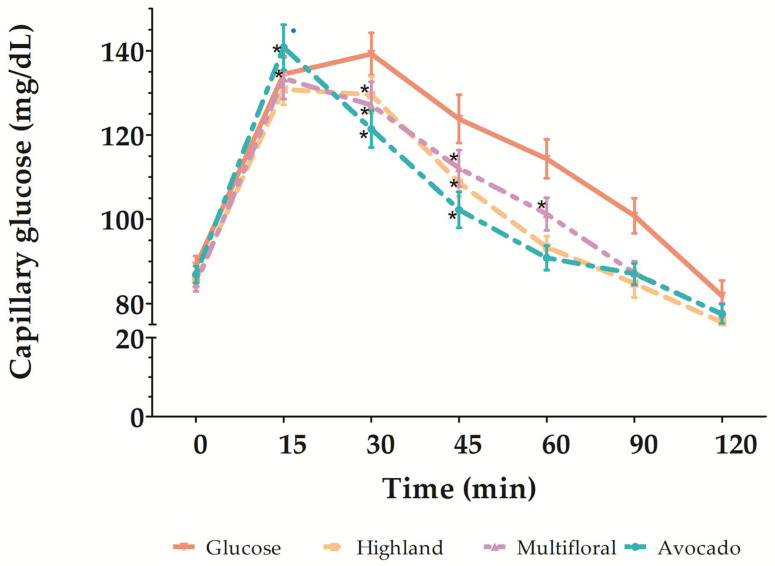
Postprandial glycemic response of honey samples. (*) Statistical difference respect to baseline value.

The GI values from highland honey were 69.20 ± 4.07; from multifloral honey, 75.24 ± 5.98; and from avocado honey, 66.36 ± 5.74 (Table 3), which did not show statistically significant differences (α ≤ 0.05). According to these results, highland and avocado honey samples resulted in a medium GI, while the multifloral honey sample showed a high GI. Although glucose presented the highest value of postprandial glucose incremental (147.46 ± 4.57 mg/dL), no significant differences were found compared with honey samples (Table 3). Figure 1 shows the postprandial glycemic response of honey samples. Highland and multifloral honey samples indicated significant differences up to 45 min from the baseline value. However, the avocado honey sample showed a significant difference from the baseline value at up to 60 min, suggesting a better postprandial glycemic response (Figure 1).

The GI and response satiety for Mexican types of honey have not been extensively investigated. To our knowledge, this is the first report about the highland, multifloral, and avocado (*Persea americana*) types of honey of Mexican origin. Honey is a natural sweetener, and its chemical composition is affected by its botanical origin and geographical region, factors that can affect glycemic response [16]. The GI results obtained in this study are similar to those reported by other authors for different varieties of honey. The results obtained for highland and avocado honey (medium GI) are similar to those obtained for clover, bhekkar, rusberry, and chestnut honeys [29,30]. On the other hand, the value of multifloral honey, classified as high GI, is similar to those found for tupelo, cotton, buckwheat, and heather honey [16,29]. However, Vadasery and Ukkuru [3] reported lower GI values for raw and processed multifloral honey from India (63 GI values for both of them) in comparison with the analyzed samples in this study (66.36–75.24).

The International Tables of GI and Glycemic Load ranks honey as medium GI, with an average value of 61 [14]. The discrepancies observed may be due to different botanical sources and geographical variables, such as soil and climatic conditions, which impact the distribution of nutrients in honey [14,16].

Although this is not clear yet, honey’s fructose content and the presence of antioxidants may influence its GI, because it has been negatively correlated with blood glucose response [31,32]. Moreover, honey’s ability to inhibit α-amylase and α-glucosidase has been reported, which also could be influenced by the GI or hypoglycemic effects of honey. The inhibitory effect on these enzymes by honey is related to their phenolic compounds [3,32]. Color is a parameter that is determined by the botanical origin of honey as well as the mineral content and the temperature at which the honey remains in the hive and post-harvest storage time [4]. In addition, dark colors in honey can be related to a higher content of antioxidants, such as phenolic compounds. Several studies have reported a high positive correlation between phenolic compounds and antioxidant activity in honey; the color of honey has a strong correlation with flavonoid content; hence, the darkest honey generally presents higher flavonoid values and therefore higher antioxidant activity [33,34]. In our study, the avocado honey sample is a dark honey which showed a medium GI; this result can be explained by a high content of phenolic compounds.

Additionally, some authors have reported that the F/G ratio determines the GI of honey; however, other authors have not found a correlation between these two factors [16,29]. Nevertheless, not only fructose could explain the glucose role in metabolism regulation but also several fractions of carbohydrates such as sucrose and oligosaccharides [16,35]. Deibert et al. [36] found that the trisaccharide melezitose significantly increased the GI value of pine honey. Gourdomichali and Papakonstantinou [16] reported that sucrose/oligosaccharides ratio, sucrose content, fructose content, and F/G ratio significantly affected the glycemic response of different types of honey; however, the authors concluded that the sucrose/oligosaccharide ratio showed the strongest influence.

### 3.4. Satiety Response

**Figure 2:**
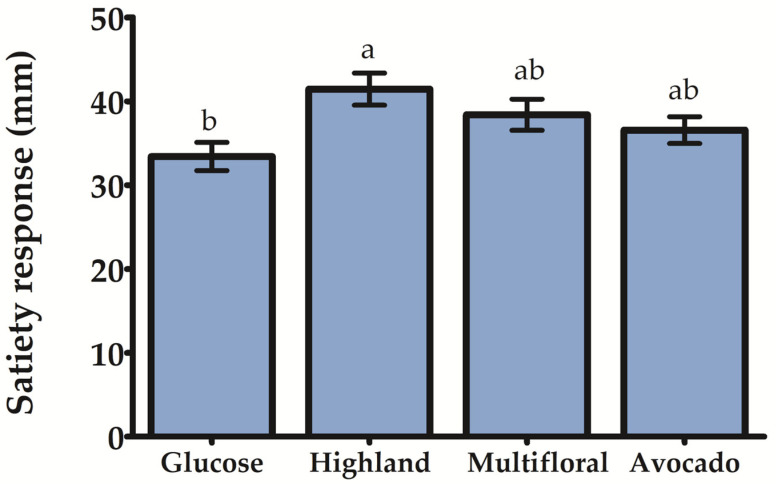
Satiety response to honey samples. The results express the mean of all satiety response values from 0 to 120 min. Different letters indicate a statistical difference, as determined via Tukey’s HSD test (α = 0.05).

Similary to the glycemic response, the satiety response was analyzed over time (Table 4). Results indicated that highland honey presented the best satiety response. Participants reported feeling satisfaction from 15 min to up to 45 min after honey consumption (α ≤ 0.05). The consumption of avocado honey caused a similar satiety response, but it was up to 30 min (α ≤ 0.05) (Table 4). Figure 2, which shows the mean value of satiety response over time, corroborates these findings since it shows that highland honey presented the highest satiety response, which was statistically significant (α ≤ 0.05) in respect to glucose. Conversely, multifloral and avocado honey presented similar satiety responses to that showed by glucose. 

The mechanism by which honey impacts satiety has not been established. Our results indicated that highland honey showed a significant effect on satiety (Figure 2), which could be considered a contribution to the body of knowledge. It has been hypothesized that high GI foods may decrease satiety due to a low fuel state occurring after consumption of these foods [8]. However, Flint et al. [37] have suggested that insulin, but not glucose, affects satiety; thus, high concentrations of insulin after consuming a meal are associated with decreased hunger and increased satiety in healthy participants [8,37]. Nevertheless, Gourdomichali and Papakonstantinou [16] showed that honey consumption did not significantly impact insulin levels or satiety in metabolically healthy subjects, but it significantly influenced the glucose response. Interestingly, Larson-Meyer et al. [15] showed that the consumption of a breakfast with honey did not increase the insulin levels, but it delayed postprandial ghrelin response, enhanced PYY response, and diminished glycemic response compared to sucrose. Recently, Hashim et al. [38] have reported that honey could normalize circulating glucose levels due to its fructose content and prolong gastric emptying, lowering food intake. Once the honey is consumed, the slow absorption of fructose within the intestinal tract might delay the interaction between fructose and the intestinal receptor, which might result in satiety. In this study, the highland honey sample showed the highest values of fructose, which could explain its higher satiety response. 

In this study, we did not quantify other carbohydrates, such as disaccharides or oligosaccharides. In order to determine the satiety effect of honey, we did not evaluate some biochemical markers, such as GLP-1, CCK, or PYY. Additionally, we did not analyze other components of honey, such as antioxidants, phenolic acids, or flavonoids. Finally, we evaluated the honey samples in a young population since the participants’ age was 23.16 ± 3.73.

However, in order to properly observe significant differences between honey samples, we used 26 participants (*n* = 26). To guarantee that participants were healthy, we analyzed several biochemical variables before they entered the study, such as fasting blood, HbA1c, total cholesterol, LDL cholesterol, VLDL cholesterol, and HDL cholesterol; in addition to this, we analyzed some anthropometric variables, such as weight and height, in order to evaluate the BMI of all participants.

## 4. Conclusions

The highland and avocado honeys showed a medium GI. Additionally, highland honey presented a higher satiety response than avocado, and multifloral honey samples. These findings could be explained by the effect of different fractions of carbohydrates present in honey, including its fructose content; however, other components such as phytochemicals might be implicated. Because of that, highland, and avocado honey might be used as a substitute for refined sugar as a natural sweetener in food. Nevertheless, further studies are needed to clear up the mechanism by which honey could affect satiety response, as well as long-term studies to determine its effect on glycemic control.

## Figures and Tables

**Table 1:** Fructose and glucose content, ratio F/G, and available carbohydrates per honey dose in highland, multifloral and avocado honey.

Sample	Fructose (g/kg)	Glucose (g/kg)	Ratio	Available Carbohydrates per Honey Doses
Highland honey	395.10 ± 0.48 ^a^	273.00 ± 0.29 ^b^	1.45	49.88 g/75 g
Multifloral honey	355.60 ± 0.15 ^b^	354.40 ± 0.00 ^a^	1.00	49.70 g/70 g
Avocado honey	272.40 ± 0.27 ^c^	232.20 ± 0.25 ^c^	1.17	49.45 g/98 g

The results express the mean of nine replicates ± standard error. Different letter in the same column indicates a statistical difference, as determined by Tukey’s HSD test (α = 0.05).

**Table 2:** Anthropometry and biochemical markers in participants.

Measurement	Value
Age (y)	23.16 ± 3.73
Glucose (mg/dL)	84.29 ± 7.00
HbA1c (%)	5.4 ± 0.22
Cholesterol (mg/dL)	149.44 ± 19.96
Triglycerides (mg/dL)	68.40 ± 29.88
HDL (mg/dL)	53.77 ± 12.65
LDL (mg/dL)	82.01 ± 17.22
VLDL (mg/dL)	13.07 ± 5.13
Weight (kg)	61.12 ± 11.69
Height (m)	1.62 ± 0.07
BMI	23.19 ± 3.07

The results express the mean ± standard error, *n* = 26.

**Table 3:** Area under curve, glycemic index and postprandial incremental glucose for glucose and highland, multifloral and avocado honey.

Parameter	Glucose	Highland Honey	Multifloral Honey	Avocado Honey
Area under curve (AUC)	224.68 ± 18.48 ^a^	155.50 ± 9.16 ^b^	169.06 ± 13.44 ^b^	149.10 ± 12.90 ^b^
Glycemic index	-	69.20 ± 4.07 ^a^	75.24 ± 5.98 ^a^	66.36 ± 5.74 ^a^
Postprandial incremental glucose (mg/mL)	147.46 ± 4.57 ^a^	140.19 ± 3.56 ^a^	141.53 ± 4.56 ^a^	143.53 ± 5.22 ^a^

The results are expressed as the mean ± standard error, *n* = 26. A different letter in the same column indicates a statistical difference, as determined via Tukey’s HSD test (α = 0.05).

**Table 4:** Satiety response for glucose, highland, multifloral, and avocado honey after intake over time (0–120 min).

Time (min)	Glucose (mm)	Highland Honey (mm)	Multifloral Honey (mm)	Avocado Honey (mm)
0	29.54 ± 4.05 ^abc^	29.42 ± 4.99 ^bc^	33.92 ± 4.74 ^ab^	28.77 ± 3.63 ^bc^
15	45.46 ± 4.84 ^a^	50.65 ± 4.67 ^a^	48.69 ± 4.53 ^a^	46.73 ± 4.34 ^a^
30	44.81 ± 4.58 ^a^	54.46 ± 4.68 ^a^	45.50 ± 4.92 ^a^	46.81 ± 4.14 ^a^
45	41.00 ± 4.06 ^ab^	52.50 ± 4.68 a	43.58 ± 4.42 ^a^	40.35 ± 4.15 ^ab^
60	32.62 ± 3.63 ^abc^	46.08 ± 5.07 ^ab^	42.31 ± 4.90 ^ab^	36.50 ± 4.23 ^abc^
90	26.88 ± 3.19 ^bc^	35.23 ± 4.05 ^abc^	32.69 ± 4.92 ^ab^	32.04 ± 4.12 ^abc^
120	16.73 ± 2.75 ^c^	20.54 ± 3.48 ^c^	22.69 ± 4.47 ^b^	23.08 ± 4.03 ^c^

The results express the mean ± standard error (*n* = 26). Different letters in the same column indicate a statistical difference, as determined via Tukey’s HSD test (α = 0.05).

## Data Availability

The data will be made available upon responsible request.
